# The Uses of 2-Ethoxy-(4*H*)-3,1-benzoxazin-4-one in the Synthesis of Some Quinazolinone Derivatives of Antimicrobial Activity

**DOI:** 10.3390/ph4071032

**Published:** 2011-07-14

**Authors:** Maher A. El-Hashash, Khalid M. Darwish, Sameh A. Rizk, Fakhry A. El-Bassiouny

**Affiliations:** 1 Chemistry Department, Faculty of Science, Ain Shams University, Abbassia, Cairo, Egypt; 2 Chemistry Department, Science Faculty, University of Garyounis, Benghazi, Libya

**Keywords:** benzoxazinone, quinazolinone, aminothiadiazole, nucleoside, antimicrobial

## Abstract

The behavior of 2-ethoxy-(4*H*)-3,1-benzoxazin-4-one (**1**) towards nitrogen nucleophiles, e.g. ethanolamine, aromatic amines (namely: *p*-toluidine, *p*-anisidine, *p*-hydroxyaniline, *o*-hydroxyaniline, *o*-bromoaniline, *o*-phenylenediamine, *p*-phenylenediamine, *o*-tolidinediamine) *p*-aminobenzoic acid, glucosamine hydrochloride, 2-aminonicotinic acid, 1-naphthalenesulfonic acid hydrazide, *n*-decanoic acid hydrazide, benzoic acid hydrazide, semicarbazide, aminoacids (e.g. D,L-alanine, L-asparagine, L-arginine) and derivatives of 2-aminothiodiazole has been investigated. The behavior of the benzoxazinone towards a selected sulfur nucleophile, L-cysteine, has also been discussed. Formation of an amidine salt as a reaction intermediate has been assumed. The effect of solvent in some reactions has been elucidated. The structures of all the novel quinazoline and quinazolinone derivatives, obtained by heterocyclic ring opening and ring closure were inferred by the IR, MS as well as ^1^H-NMR spectral analysis. Moreover, the antimicrobial potential of some of the new synthesized derivatives has been evaluated.

## 1. Introduction

**Scheme 1:**
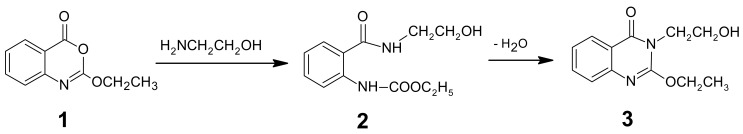
Synthetic pathway for compounds **2** and **3**.

3,1-Benzoxazin-4-ones can be considered as semiacid anhydrides which undergo many of the reactions of true acid anhydrides, but at a slower rate. This special reactivity allows this class of compounds to be useful as antimicrobial [1], anti-platelet aggregation [2], human leukocyte elastase inhibitors [3], receptor agonist active [4], receptor antagonist active [5-9], pesticides [10], tissue culture protective and *in vivo* model of neurodegeneration [11] and improve the umbilical vein endothelial cells [12]. In this paper, we report both the uses of 2-ethoxy-(4*H*)-3,1-benzoxazin-4-one (1, Scheme 1) in the synthesis of some quinazolinone derivatives and the screening of antimicrobial activity of some of the newly synthesized derivatives against Gram-negative and Gram-positive bacteria as well as fungi by means of the disc diffusion method.

## 2. Results and Discussion

Herein we report the behavior of benzoxazinone derivative **1** towards some nitrogen and sulfur nucleophiles with the aim of obtaining more precise information about the course of the reaction (the ethoxy group has a small size and negative inductive effect). Thus the reaction of derivative **1** with ethanolamine in boiling ethanol yielded compound **2** (Scheme 1) which on heating above its melting point (120-121 °C) yielded the desired product **3**.

**Scheme 2:**
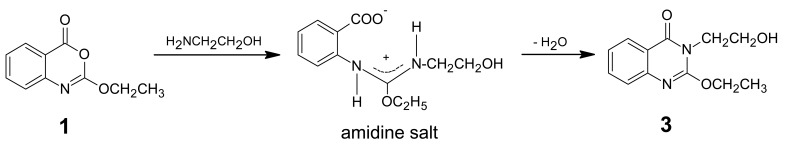
Formation of the amidine salt of compound **3**.

The elemental analyses and spectroscopic data for **2** and **3** are consistent with the assigned structures. Isolation of product **2** ruled out the abnormal nucleophilic addition to C-2 to form the amidine salt which subsequently dehydrates to give the desired product **3** [12] (Scheme 2):

The mass spectrum for product **3** showed a molecular ion peak at *m/z* 234, 236 for the parent compound followed by ions at *m/z* 190,192 and *m/z* 174,176 attributable for 2-ethoxyquinazolin-4-one and 2-ethoxyquinazoline, respectively.

**Scheme 3:**
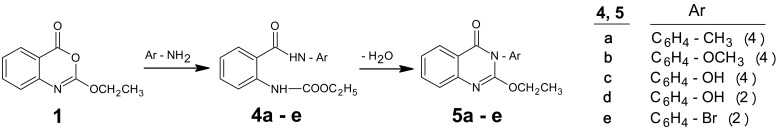
Synthetic pathway for compounds **4** and **5**.

Reacting compound **1** with aromatic amines namely, *p*-toluidine, *p*-anisidine, *p*-hydroxyaniline, *o*-hydroxyaniline and *o*-bromoaniline in boiling ethanol afforded the corresponding **4a-e** in good yields. These, in turn, can be cyclized to the corresponding quinazolinones **5a-e** under thermal conditions (240-260 °C) or with acetic anhydride. The formation of compound **4** possibly takes place via heteroring opening via nucleophilic addition at the more reactive C-4 in the oxazinone moiety (no ring closure takes place under this condition due to the small size of ethoxy group that does not enhance cyclization), but under thermal conditions ring closure was obtained (Scheme 3).

**Scheme 4:**
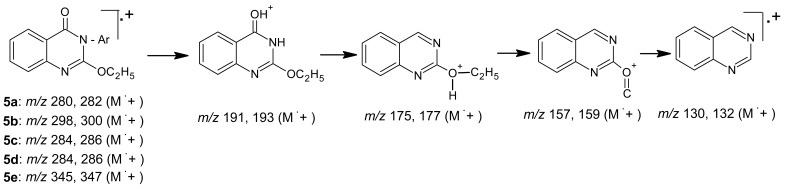
Mass spectra interpretation for compounds **5a-e**.

Interpretation of the mass spectra for products **5a-e** always showed molecular ion peaks of the parent compounds, followed by ion peaks for further fragments, including those for 2-ethoxy-4-(*3H*)-oxoquinazoline at *m/z* 191,193 which could be attributed for the loss of sugar residue, and ending with those for the 2-ethoxyquinazoline at *m/z* 175, 177 (Scheme 4).

**Scheme 5:**
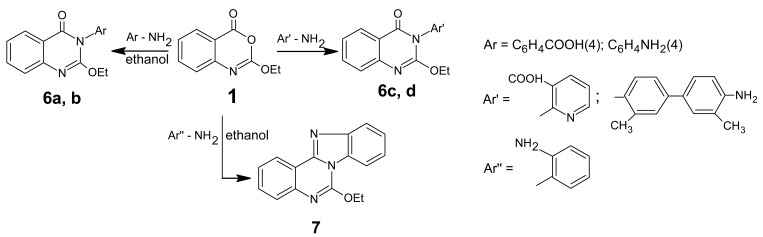
Synthetic pathways for compounds **6-7**.

Reacting compound **1** with *p*-aminobenzoic acid (in boiling butanol) and *p*-phenylenediamine (in boiling ethanol) afforded quinazolones **6a** and **6b** respectively. Also, reacting compound **1** with 2-aminonicotinic acid and 3,3′-dimethyl-4,4′-biphenyldiamine (*o*-tolidinediamine) each in boiling ethanol afforded quinazolinones **6c** and **6d**, respectively. But when compound **1** was reacted with *o*-phenylenediamine in boiling ethanol it yielded the tetracyclic product **7.** The structures of products **6–7** were based on the microanalytical and spectral data (Scheme 5).

**Scheme 6:**
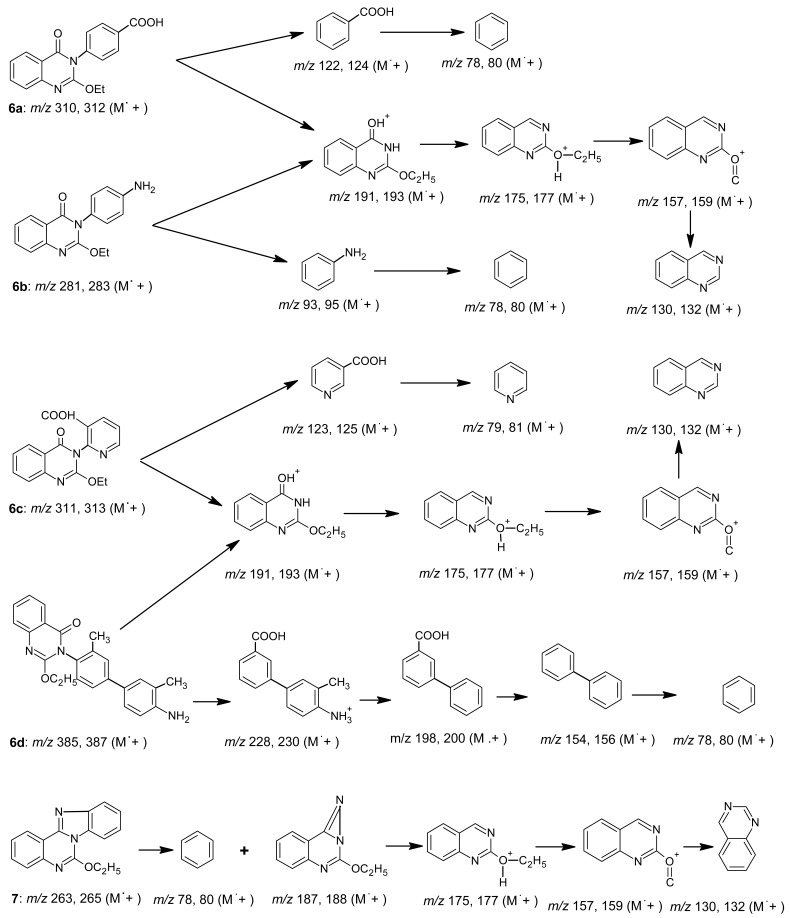
Mass spectra interpretation for compounds **6a-d** and **7**.

Interpretation for the mass spectra for compounds **6a-d** and **7** is shown in Scheme 6.

**Scheme 7:**
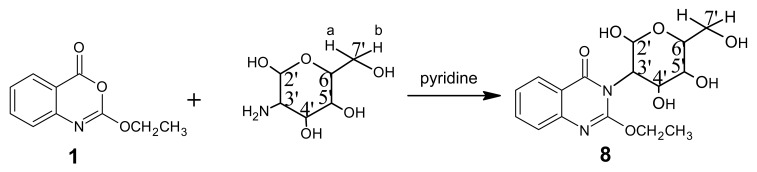
Synthetic pathway for compound **8**.

It is well known that cyclic and acyclic nucleosides often enhance the biological activity of heterocyclic derivatives [11]. Thus, product **1** when glycosidated by glucosamine hydrochloride in the presence of pyridine, afforded the quinazolinone derivative **8** (Scheme 7). The structure of compound **8** was established according to its microanalytical and spectroscopic data.

**Scheme 8:**
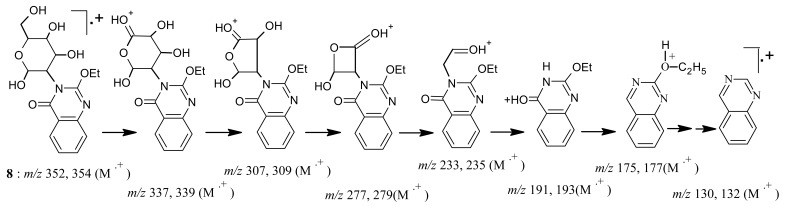
Mass spectrum interpretation for compound **8**.

The mass spectrum interpretation for compound **8** is interpreted in Scheme 8.

**Scheme 9:**
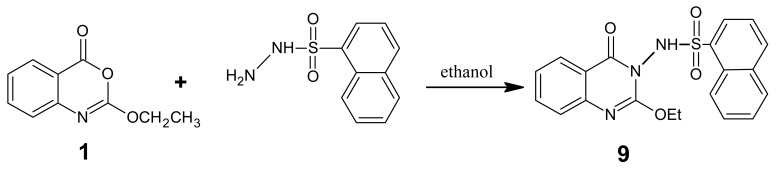
Synthetic pathway for compound **9**.

Sulfonamides are well known for their interesting antibacterial and antifungal activities [13]. Therefore, product **1** when reacted with *o*-naphthalenesulonyl hydrazide in boiling ethanol gave product **9** (Scheme 9).

**Scheme 10:**
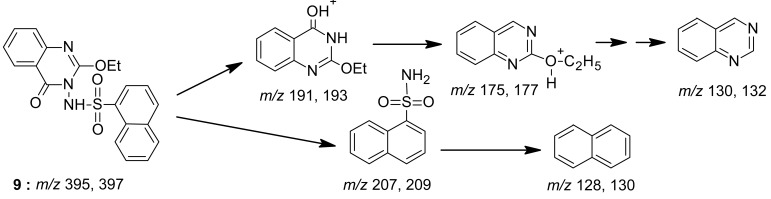
Mass spectrum interpretation for compound **9**.

The mass spectrum interpretation for compound **8** is shown in Scheme 10.

**Scheme 11:**
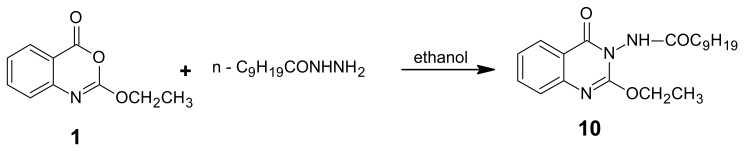
Synthetic pathway for product **10**.

The reaction of *n*-decanohydrazide with product **1** in ethanol gave product **10** (Scheme 11).

**Scheme 12:**
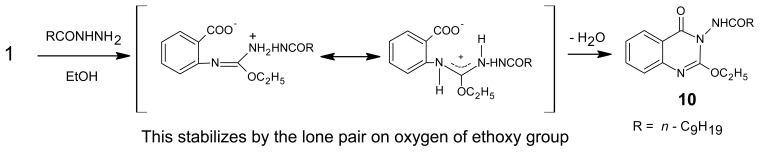
Formation of amidine salt of compound **10**.

The reaction possibly took place via hydrazide hydrogen that bonded to the nitrogen atom of the heterocycle then underwent an “abnormal” nucleophilic attack to C-2 to form the amidine salt which subsequently dehydrated to give the product (Scheme 12). The structure of product **10** was based on the microanalytical and spectral data.

**Scheme 13:**
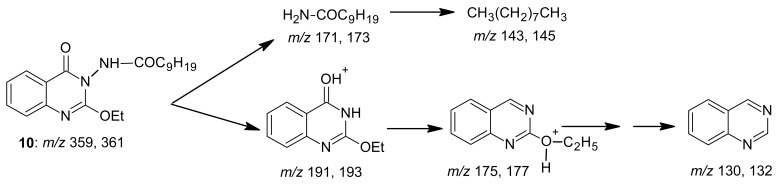
Mass spectrum interpretation for compound **10**.

An interpretation of the mass spectrum data for compound **10** is shown in Scheme 13.

**Scheme 14:**
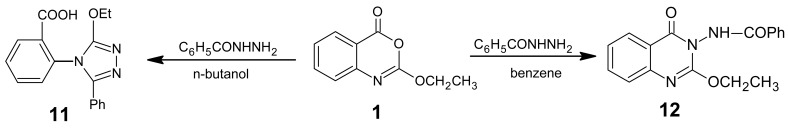
Synthetic pathway for compounds **11** and **12**.

The outcome of the reaction of compound **1** with benzohydrazide depended on the solvent used. For instance, when the reaction was carried out in *n*-butanol derivative **11** was obtained, whereas when the reaction ocurred in benzene derivative **12** was obtained (Scheme 14).

**Scheme 15:**
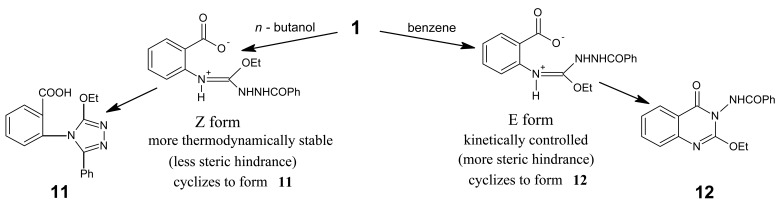
Stability of amidine salts forming compounds **11** and **12**.

**Scheme 16:**
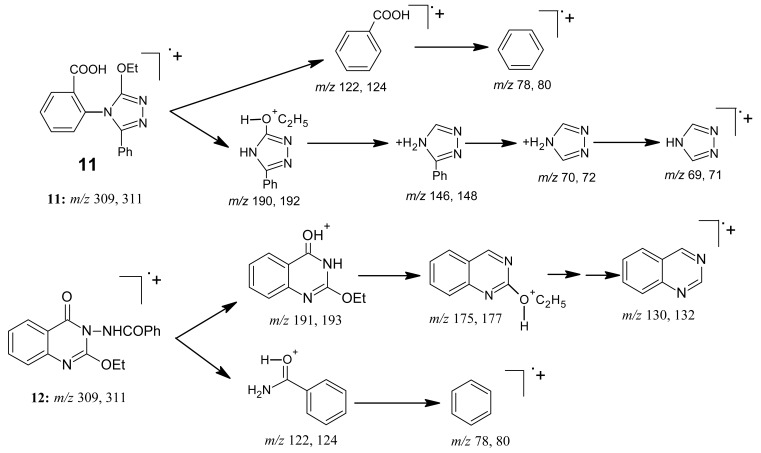
Mass spectra interpretation for compounds **11** and **12**.

This revealed that in *n*-butanol the reaction took place via the heteroring opening at C-2 followed by cyclisation to give product **11**, where no quinazolone derivative was obtained due to the amidine salt present in the more thermodynamically stable Z-form (Scheme 15), whereas in benzene the product **12** (as the kinetically controlled product) was obtained. However, carrying out this reaction in ethanol, instead, increases the difficulty for ring closure. This might be because of the lower boiling point of ethanol (78 °C), as a protic solvent, compared with that of water (100 °C), whereas benzene (b.p. 80 °C) is aprotic. The structures of products **11** and **12** were based on microanalytical and spectral data. Interpretation of the mass spectral data is shown in Scheme 16.

**Scheme 17:**
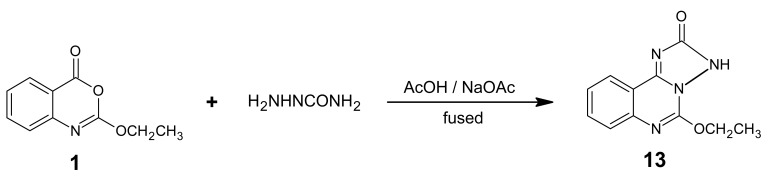
Synthetic pathway for compound **13**.

**Scheme 18:**
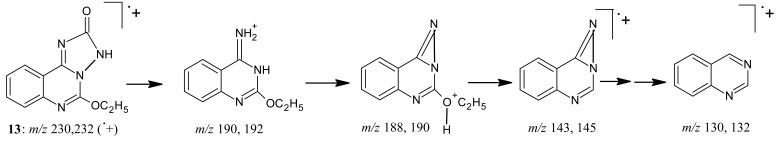
Mass spectrum interpretation for compound **13**.

Heating of compound **1** with semicarbazide in acetic acid in the presence of fused sodium acetate gave compound **13** (Scheme 17). The reaction took place via heteroring opening at C-4 then dehydration. The structure of product **13** was based on microanalytical and spectral data. The mass spectrum interpretation is shown in Scheme 18.

**Scheme 19:**
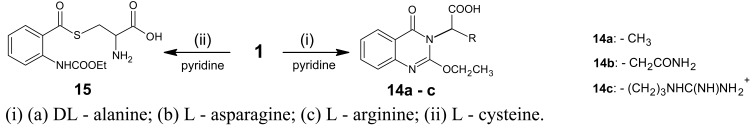
Synthetic pathway for compounds **14** and **15**.

Benzoxazinone **1** when reacted with amino acids, namely D,L-alanine, L-asparagine, and L-arginine under fusion conditions at 190 °C or by refluxing in pyridine in the presence of few drops of water produced derivatives **14a-c**, respectively [14-16] (Scheme 19). The reaction took place via heteroring ring opening at C-4 followed by water elimination. In contrast, the reaction of L-cysteine with compound **1** in boiling pyridine yielded compound **15**, where the reaction took place via the heteroring opening at C-4 by the sulfur nucleophile (rather than nitrogen nucleophile) to give derivative **15** as an *S*-aroylcysteine. The *S*-substituted cysteines are either products or used as intermediates in many syntheses [17]. The structures of products **14a-c** and **15** were based on microanalytical and spectral data.

**Scheme 20:**
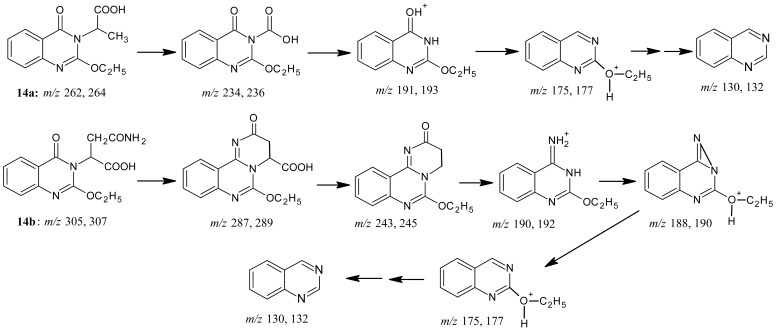

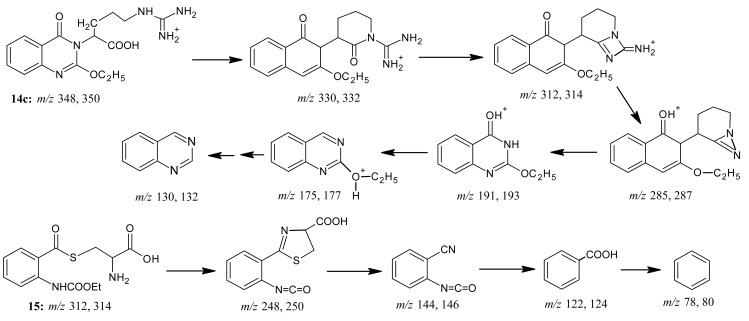
Mass spectra interpretation for compounds **14** and **15**.

The mass spectra interpretation for compounds **14a-c** and **15** is shown in Scheme 20.

Reacting benzoxazinone **1** with heteroaromatic amines, namely 2-phenyl-5-aminothiadiazole, 2-cinnamyl-5-aminothiadiazole, or 5-phthalimidomethyl-2-aminothiadiazole in boiling acetic acid in the presence of fused sodium acetate afforded 2-ethoxy-3- substituted-quinazolones **16a-c** (Scheme 21). The structures of products **16a-c** were based on microanalytical and spectral data. Interpretation for the mass spectra of derivatives **16a-c** is shown in Scheme 22.

## 3. Antimicrobial Evaluation

Compounds **3**, **6b**, **6d**, **8**, **9**, **10**, **12**, **14**, **16a**, **16b** and **16c** were tested for antimicrobial activity against *Escherichia coli* (Gram negative bacterium), *Staphylococcu*s *aureus* (Gram positive bacterium), *Aspergillus flavus* and *Candida albicans* (fungi) using the disc diffusion method. The antimicrobial evaluation was done in the Microanalytical Center at Cairo University.

### 3.1. General disc diffusion (agar-based) method

Standard discs of tetracycline (antibacterial agent) and amphotericin B (antifungal agent) served as positive controls and references for antimicrobial activities respectively, but filter discs impregnated with 10 μL of solvent (chloroform, ethanol, DMF) were used as a negative control. The agar used is Meuller– Hinton agar that is rigorously tested for composition and pH. The depth of the agar in the plate is a factor to be considered in this method. Blank paper discs (Schleicher and Schuell, Spain) with a diameter of 8.0 mm were impregnated with 10 μL of the tested concentration of the stock solutions. When a filter paper disc impregnated with a tested chemical is placed on agar, the chemical will diffuse from the disc into the agar. This diffusion will place the chemical in the agar only around the disc. The solubility of the chemical and its molecular size will determine the size of the area of chemical infiltration around the disc. If an organism is placed on the agar it will not grow in the area susceptible to the chemical around the disc. This area of no growth around the disc is the “zone of inhibition” or “clear zone”. For disc diffusion, the zone diameters were measured with slipping calipers of the National Committee for Clinical Laboratory Standards (NCCLS) [18]. Agar-based method is a good alternative method being simpler and faster than broth–based methods [19, 20].

## 4. Antibacterial Activity

Results of antibacterial activity tested against *Escherichia coli* (G-) and *Staphylococcus aureus* (G+) showed that all of the selected compounds are antibacterially active and comparatively efficient.

## 5. Antifungal Activity

Results of antifungal activity tested against two strains of fungi namely, *Aspergillus flavus* and *Candida albicans* showed that compounds **9**, **16b** and **16c** were active against both fungi, compound **16a** was active only with *A. flavus* and compounds **6b**, **8**, **10** and **12** were only active on *C. albicans*, whereas the rest of compounds were inactive.

**Figure 1:**
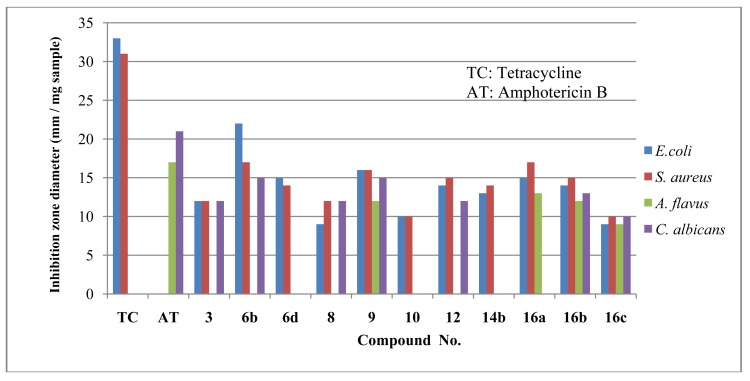
Graphical representation of the antimicrobial activity of tested compounds compared to tetracycline and amphotericin B.

In conclusion all the compounds **3**, **6b**, **6d**, **8**, **9**, **10**, **12, 14b** and **16a-c** were antibacterially active and comparatively efficient. In addition, compounds **9**, **16b and 16c** were active against both fungi, **16a** was active only with *A. flavus*, **6b**, **8**, **10** and **12** were active only with *C. albicans*, and the rest were inefficient. The following graph (Figure 1) represents the antimicrobial activity of these products compared to those of tetracycline and Amphotericin B.

## 6. Experimental

### 6.1. General

All melting points recorded are uncorrected. The IR spectra were recorded on a Pye Unicam SP1200 spectrophotometer using the KBr wafer technique. The ^1^H-NMR spectra were determined on a Varian FT-200 or Bruker AC-200 MHz instrument using TMS as an internal standard. Chemical shifts (δ) are expressed in ppm. The mass spectra were determined using MP model NS-5988 and Shimadzu single focusing mass spectrometer (70 eV). All solvents used were of HPLC/AnalaR grade. All reagents were used as received from Alfa Aesar. Compound **1** was prepared according to methods available in the literature [21], and in the order to avoid moisture it was immediately used after preparation, prior to each synthesis.

### 6.2. General Procedure for the Synthesis of Compounds **2** and **3**

A mixture of 2-ethoxy(4*H*)-3,1-benzoxazin-4-one **1** (0.01 mol) and ethanolamine (0.01 mol) in boiling ethanol (30 mL) was refluxed for 3 h. Concentration of the solvent left a white precipitate of compound **2** which was crystallized from ethanol affording beige white crystals. Heating product **2** above its melting point yielded the corresponding product **3.**

*2- Ethoxycarbonylamino(β-hydroxyethyl)benzamide* (**2**): Yellowish white crystals from ethanol; m.p. 120-121 °C; yield 80%; Anal. for C_12_H_16_N_2_O_4_ (m.w. 252); Found: C, 57.21; H, 6.29; N, 11.13; Calcd: C, 57.14; H, 6.35; N, 11.11; IR υ (cm^-1^) 1636 (C=O), 1737 (C=O), 3069 (CH), 3130 (NH), 3342 (OH); MS: *m/z* [252]^+^.

*2-Ethoxy–3-(2-hydroxyethyl)quinazolin–4–one* (**3**). Light brown crystals from ethanol; m.p. 108–109 °C; yield 75%; Anal. for C_12_H_14_N_2_O_3_ (m.w. 234); Found: C, 61.05; H, 5.98; N, 12.03; Calcd: C, 61.54; H,5.98; N, 11.97; IR υ (cm^-1^) 1660 (C=O), 3340 (OH). MS: *m/z* (int. %) [M+H]^+^ 234 (58.0), 236 (12.8), 190 (100), 192 (22.3); 174 (22.3), 176 (12.4); ^1^H-NMR (DMSO-d_6_) δ1.19 (t, 3H; -OCH_2_*CH_3_, J* =7.4 Hz), 3.52 (m, 2H, 2′-H), 4.32 (q, 2H; -O*CH_2_*CH_3_*, J* =7.4 Hz), 4.13 (m, 1H, 1′-H), 5.72 (s, br., OH), 7.41−8.16 (4 d, 4H; ArH).

### 6.3. General procedure for the synthesis of compounds **4a-e**

A mixture of benzoxazinone **1** (0.01 mol) and an aromatic amine, namely *p*-toluidine, *p*-anisidine, *p*-hydroxyaniline, *o*-hydroxyaniline and *o*-bromoaniline (0.01 mol), in boiling ethanol (40 mL) was refluxed for 3-6 h. The obtained precipitate was filtered off, washed with water, dried and crystallized from proper solvent to give the corresponding products **4a-e**, respectively.

*2-Ethoxycarbonylamino–N-(4-methylphenyl)benzamide* (**4a**): Brown crystals from ethanol; m.p. 116-117 °C; yield 85%; Anal. for C_17_H_18_N_2_O_3_ (m.w. 298); Found: C, 68.68; H, 6.12; N, 9.40; Calcd: C, 68.46; H, 6.04; N, 9.40; IR υ (cm^-1^) 1636, 1726 (2×C=O), 3361 (NH). MS: *m/z* (int. %) [M+H]^+^ 298; ^1^H-NMR (DMSO-d_6_) δ 1.28 (t, 3H; -OCH_2_*CH_3_, J* = 7.4Hz), 2.21 (s, 3H; ArCH_3_), 4.16 (q, 2H; -O*CH_2_*CH_3_*, J* = 7.4 Hz), 7.08-7.18 (m, 4H; Ph-H), 6.99–7.67 (m, 4H, quinazolinone), 9.92 (s, 1H, amide NH), 10.76 (s, 1H, amino, D_2_O exchangeable).

*2-Ethoxycarbonylamino–N-(4-methoxyphenyl)benzamide* (**4b**): Brown crystals from ethanol; m.p. 133–134 °C; yield 80%; Anal. for C_17_H_18_N_2_O_4_ (m.w. 314); Found: C, 64.88; H, 5.64; N, 8.89; Calcd: C, 64.97; H, 5.73; N, 8.91; IR υ (cm^-1^) 1638, 1725 (2×C=O), 3361 (NH). MS: *m/z* (int. %)[M+H]^+^ 314 (68.2%).

*2-Ethoxycarbonylamino-N-(4-hydroxyphenyl)benzamide* (**4c**): Brown crystals from ethanol; m.p. 114–115 °C; yield 75%; Anal. for C_16_H_16_N_2_O_4_ (m.w. 300); Found: C, 64.06; H, 5.28; N, 9.21; Calcd: C, 64.00; H, 5.33; N, 9.33; IR υ (cm^-1^) 1640, 1734 (2×C=O), 3321 (NH), 2992 (OH).

*2-Ethoxycarbonylamino-N-(2-hydroxyphenyl)benzamide* (**4d**): Brown crystals from ethanol; m.p. 111-112 °C; yield 75%; Anal. for C_16_H_16_N_2_O_4_ (m.w. 300); Found: C, 64.02; H, 5.30; N, 9.23; Calcd: C, 64.00; H, 5.33; N, 9.33; IR υ (cm^-1^) 1638, 1713 (2×C=O), 3268 (NH), 3402 (OH). MS: *m/z* (int. %) [M+H]^+^ 300 (44%).

*2-Ethoxycarbonylamino-N-(2-bromophenyl)benzamide* (**4e**): Brown crystals from ethanol; m.p. 105-106 °C; yield 85%; Anal. for C_16_H_15_BrN_2_O_3_ (m.w. 363); Found: C, 52.77; H, 4.16; N, 7.72; Br, 22.16; Calcd: C, 52.89; H, 4.13; N, 7.71; Br, 22.04; IR υ (cm^-1^) 1637, 1740 (2×C=O), 3402 (NH); MS: *m/z* (int.%) [M+H]^+^ 363 (47.4%).

### 6.4. General procedure for the synthesis of compounds **5a-e**

*Method A*: Compounds **4a-e** (0.01 mol) were heated in a round bottom flask (25 mL) in an oil bath at 160 °C for 30 minutes. After cooling the products were crystallized from the proper solvent to give the corresponding quinazolinones **5a-e**, respectively.

*Method B*: A solution of compound **4a-e** (0.01 mol) in acetic anhydride (15 mL) was heated under reflux for 2 hr. The solid that separated out after dilution with water was filtered off, dried and crystallized from the proper solvent to give compounds **5a-e**, respectively.

*2-Ethoxy-3-(4-methylphenyl)quinazolin-4-one* (**5a**): Dark brown crystals from light petroleum (100–120 °C); m.p. 91–92 °C; yield 90%; Anal. for C_17_H_16_N_2_O_2_ (m.w. 280); Found: C, 72.34; H, 5.62; N, 9.89; Calcd: C, 72.34; H, 5.67; N, 9.93; IR υ (cm^-1^) 1670 (C=O); MS: *m/z* (int. %) [M+H]^+^ 280 (58.0), 282 (13.8), 191 (100), 193 (18.2), 175 (8.3), 177 (0.7), 157 (6.3), 159 (0.6), 130 (67.0), 132 (11.9); ^1^H-NMR (DMSO-d_6_) δ 1.24 (t, 3H; -OCH_2_*CH_3_*, *J* = 7.4 Hz), 2.21 (s, 3H; ArCH_3_), 4.18 (q, 2H; -O*CH_2_*CH_3_, *J* = 7.4 Hz), 7.40–7.60 (m, 4H; Ph-H), 7.08–7.18 (m, 4H, quinazolinone).

*2-Ethoxy-3-(4-methoxyphenyl)quinazolin-4-one* (**5b**): Dark brown crystals from toluene; m.p. 114–115 °C; yield 85%; Anal. for C_17_H_16_N_2_O_3_ (m.w. 298); Found: C, 68.32; H, 5.32; N, 9.41; Calcd: C, 68.46; H, 5.37; N, 9.40; IR υ (cm^-1^) 1668 (C=O); MS: *m/z* (int. %) [M+H]^+^ 298 (52.3), 300 (31.1), 191 (100), 193 (13.5), 175 (27.2), 177 (0.7), 157 (3.3), 159 (0.4), 130 (54.6), 132 (8.3); ^1^H-NMR (DMSO-d_6_) δ 1.22 (t, 3H; -OCH_2_*CH_3_*, *J* = 7.4 Hz), 3.76 (s, 3H; OCH_3_), 4.35 (q, 2H; -O*CH_2_*CH_3_, *J* = 7.4 Hz), 6.66–7.68 (m, 4H; Ph-H), 7.47–8.19 (m, 4H, quinazolinone).

*2-Ethoxy-3-(4-hydroxyphenyl)quinazolin-4-one* (**5c**): Dark brown crystals from benzene; m.p. 105–106 °C; yield 80%; Anal. for C_16_H_14_N_2_O_3_ (m.w. 284); Found: C, 67.42; H, 4.70; N, 9.81; Calcd: C, 67.60; H, 4.93; N, 9.86; IR υ (cm^-1^) 1671 (C=O), 2992 (OH); MS: *m/z* (int. %) [M+H]^+^ 284 (43.0), 286 (16.3), 191 (100), 193 (52.3), 175 (20.8), 177 (0.1), 157 (4.3), 159 (0.5), 130 (57.4), 132 (7.9); ^1^H-NMR (DMSO-d_6_) δ 1.22 (t, 3H; -OCH_2_*CH_3_*, *J* = 7.4Hz), 4.35 (q, 2H; -O*CH_2_*CH_3_, *J* = 7.4 Hz), 5.35 (s, H; OH), 6.68–7.69 (m, 4H; Ph-H), 7.43–8.19 (m, 4H, quinazolinone).

*2-Ethoxy-3-(2-hydroxyphenyl)quinazolin-4-one* (**5d**): Dark brown crystals from light petroleum (100–120 °C); m.p. 89-90 °C; yield 80%; Anal. for C_16_H_14_N_2_O_3_ (m.w. 284); Found: C, 67.31; H, 4.73; N, 9.89; Calcd: C, 67.60; H, 4.93; N, 9.86; IR υ (cm^-1^) 1669 (C=O), 2988 (OH); MS: *m/z* (int. %) [M+H]^+^ 284 (48.0), 286 (6.3), 191 (100), 193 (43.3), 175 (18.2), 177 (0.1), 157 (4.7), 159 (1.2), 130 (67.1), 132 (6.3); ^1^H-NMR (DMSO-d_6_) δ 1.22 (t, 3H; -OCH_2_*CH_3_*, *J* = 7.4 Hz), 4.36 (q, 2H; -O*CH_2_*CH_3_, *J* = 7.4 Hz), 5.55 (s, H; OH), 6.76–7.67 (m, 4H; Ph-H), 7.30–8.19 (m, 4H, quinazolinone).

*2-Ethoxy-3-(2-bromophenyl)quinazolin-4-one* (**5e**): Dark brown crystals from light petroleum (80–100 °C); m.p. 93–94 °C; yield 90%; Anal. for C_16_H_13_BrN_2_O_2_ (m.w. 345); Found: C, 67.31; H, 4.73; N, 9.89; Br, 23.25; Calcd: C, 67.60; H, 4.9; N, 9.86; Br, 23.19; IR υ (cm^-1^) 1670 (C=O); MS: *m/z* (int. %) [M+H]^+^ 345 (66.0), 347 (22.4), 191 (100), 193 (28.3), 175 (23.3), 177 (0.2), 157 (5.8), 159 (0.4), 130 (61.4), 132 (5.4); ^1^H-NMR (DMSO-d_6_) δ 1.21 (t, 3H; -OCH_2_*CH_3_*, *J* = 7.4 Hz), 4.36 (q, 2H; -O*CH_2_*CH_3_, *J* = 7.4 Hz), 7.26-7.51 (m, 4H; Ph-H), 7.29-8.19 (m, 4H, quinazolinone). Compounds **5a-e** were devoid any ester and/or NH group bands.

### 6.5. General Procedure for the Synthesis of Compounds **6a-d**

A mixture of benzoxazinone **1** (0.01 mol) and any of the aromatic amines *p*-aminobenzoic acid, 2-aminonicotinic acid, *p*-phenylenediamine, or 3,3′-dimethyl-4,4′-biphenyldiamine (0.01 mol) in boiling butanol (30 mL) was refluxed for 3-6 h (depending on the nucleophile). Concentrating the solution gave a solid which was washed, filtered, dried and then recrystallized from the proper solvent affording the desired quinazolinone derivatives **6a-d**, respectively.

*4-[2-Ethoxy-4-quinazolon-3-yl]benzoic acid* (**6a**): Light brown crystals from ethanol; m.p. 151–152 °C; yield 80%; Anal. for C_17_H_14_N_2_O_4_ (m.w. 310); Found: C, 65.44; H, 4.72; N, 9.00; Calcd: C, 65.80; H, 4.52; N, 9.03; IR υ (cm^-1^) 1675, 1705 (2×C=O), 3355 (chelated OH); MS: *m/z* (int. %) [M+H]^+^ 310 (63.0), 312 (28.2), 191 (100), 193 (27.8), 175 (34.3), 177 (0.6), 130 (59.3), 132 (0.2), 122 (1.8),124 (0.2), 78 (0.1), 80 (0.1); ^1^H-NMR (DMSO-d_6_) δ1.22 (t, 3H; -OCH_2_*CH_3_*, *J* = 6.9 Hz), 4.36 (q, 2H; -O*CH_2_*CH_3_, *J* = 6.9 Hz), 7.40-8.00 (m, 4H; Ph-H), 7.43-8.19 (m, 4H, quinazolinone), 10.6 (1H, acid proton).

*2-Ethoxy-3-(4-aminophenyl)quinazolin-4-one* (**6b**): Light blue crystal from ethanol; m.p. 105–106 °C; yield 80%. Anal. for C_16_H_12_N_3_O_2_ (m.w. 278); Found: C, 69.22; H, 4.16; N, 15.08; Calcd: C, 69.06; H, 4.32; N, 15.10; IR υ (cm^-1^) 1635 (C=N), 1670 (C=O), 3225 (NH). MS: *m/z* (int. %) [M+H]^+^ 278 (55.0), 280 (18.2), 191 (100), 193 (31.7), 175 (32.1), 177 (0.8), 157 (4.7), 159 (0.6), 130 (52.4), 132 (0.3), 93 (0.9), 95 (0.1), 78 (0.2), 80 (0.1); ^1^H-NMR (DMSO-d_6_) δ 1.22 (t, 3H; -OCH_2_*CH_3_*, *J* = 6.9 Hz), 4.35 (q, 2H; -O*CH_2_*CH_3_, *J* = 6.9 Hz), 5.12 (s, 2H, NH_2_), 6.70–7.43 (m, 4H; Ph-H), 7.47–8.19 (m, 4H, quinazolinone).

*2-[2-Ethoxy-4-oxoquinazolin-384H)-yl]pyridine-3-caboxylic acid* (**6c**): Brown crystals from ethanol; m.p. 298-300 °C; yield 80%; Anal. for C_16_H_13_N_3_O_4_ (m.w. 311); Found: C, 61.58; H, 4.26; N, 13.20; Calcd: C, 61.74; H, 4.18; N, 13.50; IR υ (cm^-1^) 1634, 1704 (2×C=O), 3255 (chelated OH); MS: *m/z* (int. %) [M+H]^+^ 311 (36.0), 313 (19.5), 191 (100), 193 (28.6), 175 (61.8), 177 (7.6), 157 (5.2), 159 (1.1), 130 (58.2), 132 (0.6), 123 (0.7), 125 (0.2), 79 (0.7), 81 (0.1); ^1^H-NMR (DMSO-d_6_) δ 1.22 (t, 3H; -OCH_2_*CH_3_*, *J* = 6.9 Hz), 4.37 (q, 2H; -O*CH_2_*CH_3_, *J* = 6.9), 7.44-8.20 (m, 4H; quinazolinone), 6.97, 7.87, 8.41 (m, 3H; H-4, H-5, pyridine moiety H-6).

*2-Ethoxy-3-(3,3′-dimethyl-4-amino)biphenylquinazolin-4-one* (**6d**): Dark brown crystals from ethanol; m.p. 121-122 °C; yield 80%; Anal. for C_24_H_23_N_3_O_2_ (m.w. 385); Found: C, 74.53; H, 5.63; N, 10.79; Calcd: C, 74.80; H, 5.97; N, 10.90; IR (KBr) υ (cm^-1^) 1620 (C=N), 1675 (C=O), 3250 (NH). MS: *m/z* (int. %) [M+H]^+^ 385 (72.0), 387 (34.2), 228 (13.3), 230 (1.2), 198 (12.7), 200 (0.1), 194 (16.1), 196 (0.4), 191 (100), 193 (24.8), 175 (53.3), 177 (8.7), 157 (7.1), 159 (1.4), 154 (1.2), 156 (0.2), 130 (46.3), 132 (0.1), 78 (0.3), 80 (0.1); ^1^H-NMR (DMSO-d_6_) δ 1.22 (t, 3H; -OCH_2_*CH_3_*, *J* = 6.9 Hz), 2.21- 2.29 (s, 6H; 2ArCH_3_), 4.4 (q, 2H; -O*CH_2_*CH_3_, *J* = 6.9 Hz), 5.08 (2H, s, NH_2_), 6.90-7.80 (m, 6H; biphenyl-H), 7.33-8.19 (m, 4H, quinazolinone).

*2-Ethoxy benzimidazolo-[1,2-c]quinazoline* (**7**): A mixture of benzoxazinone **1** (0.01 mol) and *o*-phenylenediamine (0.01 mol) in boiling butanol (30 mL) was refluxed for 3 h. Concentrating the solution left a solid product which was filtered, washed, dried and crystallized from ethanol affording light blue crystals of product **7**. M.p. 191-192 °C; yield 85%. Anal. for C_16_H_13_N_3_O (m.w. 263); Found: C, 73.02; H, 4.94; N, 15.98; Calcd: C, 73.00; H, 4.94; N, 15.97; IR υ (cm^-1^) 1607 (C=N). MS: *m/z* (int. %) [M+H]^+^ 263 (66.0), 265 (17.3), 187(44.8), 189 (4.8), 175 (28.4), 177 (11.1), 157 (3.3), 159 (0.1), 130 (78.9), 132 (12.4), 78 (2.4), 80 (0.1); ^1^H-NMR (DMSO-d_6_) δ 1.22 (t, 3H; -OCH_2_*CH_3_*, *J* = 7.1Hz), 4.31(q, 2H; -O*CH_2_*CH_3_, *J* = 7.1 Hz), 7.52-7.92 (m, 4H, benzimidazole), 7.62-8.55 (2 m, 4H; quinazoline).

*2-Ethoxy-3-(β-glucopyranosyl-3-yl) quinazolin-4-one* (**8**): A mixture of benzoxazinone **1** (0.01 mol) and glucosamine hydrochloride (0.01 mol) in pyridine (30 mL) was refluxed for 3 h. The mixture was poured in to an ice / water mixture stirred leaving a white precipitate to settle down. The precipitate was washed with water, filtered, dried and then crystallized from ethanol affording white crystals of product **8**. M.p. 179–180 °C; yield 80%. Anal. for C_16_H_20_N_2_O_7_ (m.w. 352); Found: C, 54.50; H, 5.67; N, 7.99; Calcd: C, 54.54; H, 5.68; N, 7.95; IR υ (cm^-1^) 1675 (C=O), 3236 (OH bonded), 3400 (OH non-bonded). MS: *m/z* (int. %) [M+H]^+^ 352 (48.0), 354 (21.1), 337 (19.2), 339 (8.2), 307 (2.8), 309 (1.1), 277 (0.8), 279 (0.2), 233 (0.1), 235 (0.1), 191 (88.1), 193 (3.1), 175 (100), 177 (13.1), 157 (4.6), 159 (0.5), 130 (68.9), 132 (9.4); ^1^H-NMR (DMSO-d_6_) δ 1.1 (t, 3H; -OCH_2_*CH_3_*, *J* = 6.8 Hz), 3.21-4.20 (m, 6H; H-3′, H-4′, H-5′, H-6′, H-7′a, and H-7′b), 3.7 (m, 2′-OH, 4′-OH, 5′-OH), 4.35 (q, 2H; -O*CH_2_*CH_3_, *J* = 6.8 Hz), 5.0 (d, 1H; H-2′, *J* = 6.5 Hz), 4.9 (s, 7′-OH), 7.41-8.17 (m, 4H; quinazolinone).

*2-Ethoxy-3-(1-naphthylsulphonylamino)-4(3H) quinazolone* (**9**): A mixture of benzoxazinone **1** (0.01 mol) and 1-naphthalenesulfonic hydrazide (0.01 mol) in ethanol (30 mL) was heated under reflux for 3 h. The excess solvent was distilled off and the mixture was cooled down leaving a brown solid, which was recrystallized from ethanol affording white brown crystals of product **9**. M.p. 111-112 °C; yield 65%. Anal. for C_20_H_17_N_3_O_4_S (m.w. 395); Found: C, 60.90; H, 4.16; N, 10.81; S, 8.11; Calcd: C, 60.76; H, 4.30; N, 10.63; S, 8.10; IR υ (cm^-1^) 1160 (S=O), 1610 (C=N), 1675 (C=O), 3200 (NH). MS: *m/z* (int. %) [M+H]^+^ 395 (63.2), 397 (13.2), 207 (22.7), 209 (2.4), 191 (100), 193 (15.5), 175 (14.5), 177 (0.4), 157 (3.5), 159 (0.3), 130 (58.4), 132(15.6), 128 (13.6); ^1^H–NMR (DMSO-d_6_) δ 1.19 (t, 3H; -OCH_2_*CH_3_*, *J* = 6.8 Hz), 4.44 (q, 2H; -O*CH_2_*CH_3_), 7.29 – 8.20 (m, 4H, quinazolinone), 7.72 – 8.05 (m, 7H, naphthalene); 10.6 (s, NH exchangeable).

*2-Ethoxy-3-decanoylamino-4(3H) quinazolinone* (**10**): A mixture of benzoxazinone **1** (0.01 mol) and decanoic acid hydrazide (0.01 mol) in ethanol (30 mL) was refluxed for 3 h. The solvent was concentrated and the mixture left to cool. Crystallization of the product from ethanol yielded light brown crystals of product **10.** M.p. 115–116 °C; yield 75%. Anal. for C_20_H_29_N_3_O_3_ (m.w. 359); Found: C, 66.83; H, 8.12; N, 11.37; Calcd: C, 66.85; H, 8.08; N, 11.70; IR υ (cm-^1^) 1670 (C=O), 3250 (NH). MS: *m/z* (int. %) [M+H]^+^ 359 (77.0), 361 (21.3), 191 (100), 193 (22.4), 175 (13.8), 177 (0.6), 171 (2.8), 173 (0.5), 157 (3.8), 159 (0.2),143 (1.7), 145 (0.3), 130 (27.3), 132 (13.2); ^1^H-NMR (DMSO-d_6_) δ 0.87 (t, 3H; CH_3_), 1.23-1.54 (m, 14H; 7 CH_2_), 1.15 (t, 3H; -OCH_2_*CH_3_*), 2.3 (t, 2H; CH_2_CO), 4.35 (q, 2H; -O*CH_2_CH_3_*), 7.46-8.20 (m, 4H; ArH), 10.7(s, NH, exchangeable).

*2-(3-Ethoxy-5-phenyl-4H-1,2,4-triazolo-4-yl)benzoic acid* (**11**): A mixture of benzoxazinone **1** (0.01 mol) and benzoic acid hydrazide (0.01 mol) in butanol (30 mL) was refluxed for 3h. The solvent was concentrated leaving a white solid**,** which was crystallized from ethanol affording white crystals of product **11**. M.p. 101-102 °C; yield 80%. Anal. for C_17_H_15_N_3_O_3_ (m.w. 309); Found: C, 66.14; H, 4.53; N, 13.17; Calcd: C, 66.02; H, 4.85; N, 13.59; IR υ (cm^-1^) 1690 (C=O), 2616-3481 (chelated OH). MS: *m/z* (int. %) [M+H]^+^ 309 (78.0), 311 (3.8), 190 (42.5), 192 (13.1), 146 (28.6), 148 (9.1), 122 (2.2), 124 (0.2), 78 (0.5), 80 (0.1); 70 (0.3), 72 (0.1), 69 (0.2), 71 (0.1); 1H-NMR (DMSO-d_6_) δ 1.16 (t, 3H; -OCH_2_*CH_3_, J* = 7.2 Hz), 4.22 (q, 2H; -O*CH_2_*CH_3_, *J* = 7.2 Hz), 7.46 – 7.77 (m, 5H, Ph-H), 7.45 – 8.09 (m, 4H, quinazolinone), 11.2 (s, broad 1H; OH).

*2-Ethoxy-3-benzoylamino-4(3H) quinazolinone* (**12**): Refluxing the mixture of benzoxazinone **1** and benzohydrazide (0.01 mol each) in benzene (30 mL) for 3 h gave a white solid product that was filtered, washed, dried and crystallized from ethanol giving white needles of **12** of m.p. 109–110 °C; yield 85%. Anal. for C_17_H_15_N_3_O_3_ (m.w. 309); Found: C, 66.02; H, 4.93; N, 13.57; Calcd: C, 66.02; H, 4.85; N, 13.59; IR υ (cm^-1^) 1635, 1670 (2C=O), 3230 (NH). MS: *m/z* (int. %) [M+H]^+^ 309 (55.0), 311 (11.8), 191 (100), 193 (13.7), 175 (57.2), 177 (14.2), 157 (3.9), 159 (0.5), 130 (42.5), 132 (13.2), 122 (35.6), 124 (0.1); ^1^H-NMR (DMSO-d_6_) δ 1.15 (t, 3H; -OCH_2_*CH_3_, J* = 7.2 Hz), 4.44 (q, 2H; -O*CH_2_*CH_3_), 7.46−8.00 (5H, m, Ph-H) and 7.28−8.20 (m, 4H, quinazolinone ArH).

*2-Ethoxy-5-oxo-1H-1,2,4-triazolo[2,3-c] quinazoline* (**13**): A mixture of benzoxazinone **1** (0.01 mol) and semicarbazide (0.01 mol) in acetic acid/fused sodium acetate (30 mL/2 g) was refluxed for 3 h. Pouring the solution into an ice/water mixture left a white solid, which was filtered, washed, dried and recrystallized from ethanol affording white crystals of derivative **13**. M.p. 126–127 °C; yield 75%. Anal. for C_11_H_10_N_4_O_2_ (m.w. 230); Found: C, 57.39; H, 4.36; N, 24.35; Calcd: C, 57.39; H, 4.35; N, 24.35; IR υ (cm^-1^) 1671 (C=O), 3206 (bonded NH), 3320 (non-bonded NH). MS: *m/z* (int. %) [M+H]^+^ 230 (77.0), 232 (14.3), 190 (17.3), 192 (8.2), 188 (12.4), 157 (8.4), 159 (1.5), 143 (15.4), 145 (2.3), 130 (58.6), 132 (2.0); ^1^H-NMR (DMSO-d_6_) δ 1.19 (t, 3H; -OCH_2_*CH_3_, J* = 7.2 Hz), 4.43 (q, 2H; -O*CH_2_*CH_3_), 7.18- 8.25 (4Xd, 4H, ArH), 10.5 (s, NH, exchangeable).

### 6.6. General procedure for the synthesis of 2-(ethoxy) quinazolinone-3-yl-alkylacetic acids **14a-c**

*Method A*: A mixture of benzoxazinone **1** (0.01 mol) and selected amino acids namely, D,L-alanine, L-aspargine, and L-arginine (0.01 mol) was fused in an oil bath at 190 °C for 2 h. The mixture was then poured in an ice/water mixture, stirred and left allowing the white precipitate to settle down. The precipitate was filtered, washed, dried and finally crystallized from the proper solvent.

*Method B*: A mixture of benzoxazinone **1** (0.01 mol) and selected amino acids namely, D,L-alanine, L-asparagine, and L-arginine (0.01 mol) were refluxed in a pyridine (30 mL)/water (5 mL) mixture for 3 h. The mixture was then poured in an ice/water mixture, stirred and left to allow the white solid precipitate to settle down. The solid was filtered, washed, dried and finally crystallized from the proper solvent to yield compounds **14a-c**, respectively.

*2-{2-(Ethoxy)quinazolin-4-one-3-yl}propanoic acid* (**14a**): Light brown crystals from DMF; m.p. 183–184 °C; yield (70% by *Method A* and 85% by *Method B*). Anal. for C_13_H_14_N_2_O_4_ (m.w. 262); Found: C, 59.59; H, 5.30; N, 10.67; Calcd: C, 59.54; H, 5.34; N, 10.69; IR υ (cm^-1^) 1675 (C=O cyclic amide), 1700 (C=O acid), 3300 (chelated OH). MS: *m/z* (int. %) [M+H]^+^ 262 (82.0), 264 (32.1), 234 (22.7), 236 (2.8), 191 (100), 193 (22.4), 175 (9.9), 177(2.7), 157 (6.1), 159 (0.2), 130 (49.3), 132 (18.4); ^1^H-NMR (DMSO-d_6_) δ 1.20 (t, 3H; -OCH_2_*CH_3_*, *J* = 7.1 Hz), 1.28 (d, 3H; CH_3_, *J* = 6.7 Hz), 5.24 (q, 1H; methine H, *J* = 6.7 Hz), 4.35(q, 2H; -O*CH_2_*CH_3_), 7.45-8.17 (m, 4H, Ar-H), 10.5 (1H, acid-H).

*2-{2-(Ethoxy)quinazolin-4-one-3-yl}-3-carbamoyl propanoic acid* (**14b**): Light brown crystals from DMF; m.p. 241-242 °C; yield (75% by *Method A* and 85% by *Method B*). Anal. for C_14_H_15_N_3_O_7_ (m.w. 305); Found: C, 55.16; H, 4.88; N, 13.79; Calcd: C, 55.08; H, 4.92; N, 13.77; IR υ (cm^-1^) 1670, 1675, 1705 (3 × C=O amides and acid), 3200 (NH), 3350 (chelated OH). MS: *m/z* (int. %) [M+H]^+^ 305 (80.0), 307 (11.5), 287 (12.3), 289 (1.3), 243 (12.2), 245 (2.3), 190 (100), 192 (32.2), 188 (8.4), 190 (0.8), 175 (48.6), 177 (12.3), 179 (0.7), 157 (3.8), 159 (0.1), 130 (55.2), 132 (0.3); ^1^H-NMR (DMSO-d_6_) δ 1.2 (t, 3H; -OCH_2_*CH_3_*, *J* = 7.1 Hz), 3.17 (m, 2H; CH_2_), 3.0 (s, NH), 5.7 (q, H; methine H, *J* = 6.7 Hz), 4.35 (q, 2H; -O*CH_2_*CH_3_), 7.45-8.17 (m, 4H, Ar-H), 10.6 (1H, acid-H).

*2-Amino-2-{2-(ethoxy)quinazolin-4-one-3-yl}-5-guanidinopentanoic acid* (**14c**): Light brown crystals from DMF; m.p. 241-242 °C; yield (70% by *Method A,* 80% by *Method B*). Anal. for C_16_H_21_N_5_O_4_ (m.w. 347); Found: C, 55.12; H, 6.13; N, 20.16; Calcd: C, 55.33; H, 6.05; N, 20.17; IR υ (cm^-1^) 1571(NH_2_), 1613 (C=N guanidinium), 1670 (C=O cyclic amide), 1700 (C=O acid), 3181 (NH), 3350 (chelated OH). MS: *m/z* (int. %) [M+H]^+^ 348 (80.0), 350 (34.2), 330 (2.4), 332 (0.2), 312 (3.8), 314 (0.2), 285 (2.2), 287 (0.1), 191 (100), 193 (42.2), 175 (38.1), 157 (4.6), 159 (0.5), 157 (6.7), 159 (0.4), 177 (12.9), 130 (48.2), 132 (0.1); ^1^H-NMR (DMSO-d_6_) δ 1.2 (t, 3H; -OCH_2_*CH_3_*, *J* = 7.1 Hz), 1.69 (2H, m, arg. C-4), 1.95 (2H, m, arg. C-3), 3.43 (2H, t, arg. C-5), 5.39 (1H, t, arg. C-2), 4.38 (q, 2H; -O*CH_2_*CH_3_), 7.46 – 8.17 (m, 4H, ArH), 6.9-7.1 (br. d, NH_2_^+^ guanidinium grp.), 7.8 – 8.0 (br. s., guanidinium grp. NH), 10.5 (1H, acid-H).

*2-Ethoxycarbonylamino-(carboxyaminomethyl thiomethyl benzoate)* (**15**): Benzoxazinone **1** and cysteine (0.01 mol each) were refluxed in a mixture of pyridine (30 mL) / water (5mL) for 3h. The mixture was poured in an ice/water mixture, stirred and left yielding a white solid precipitate. The precipitate was filtered, washed, dried and crystallized from DMF affording white crystals of product **15.** M.p.121-122 °C; yield 85%. Anal. for C_13_H_16_N_2_O_5_ S (m.w. 312); Found: C, 50.39; H, 5.22; N, 8.88; S, 10.02; Calcd: C, 50.00; H, 5.13; N, 8.97; S, 10.26. IR υ (cm^-1^) 1670, 1735, 3223, 3340. MS: *m/z* (int. %) [M+H]^+^ 312 (82.0), 314 (48.0), 248 (28.8), 250 (11.1), 144 (12.6), 146 (2.3), 122 (0.8), 124 (0.1), 78 (0.2), 80 (0.1); ^1^H-NMR (DMSO-d_6_) δ 1.24 (t, 3H; -OCH_2_*CH_3_*, *J* = 7.2 Hz), 3.56 (d, 2H; CH_2_, *J* = 6.5 Hz), 3.63 (t, methine proton, *J* = 6.5 Hz), 4.19 (q, 2H; -O*CH_2_*CH_3_), 6.5 (1H, NH, exchangeable), 7.29-7.53 (m, 4H; ArH), 10.6 (1H, acid-H).

### 6.7 General procedure for the synthesis of 2-ethoxy-3-substituted quinazolones **16a-c**

A mixture of benzoxazinone **1** (0.01 mol) and the aminothiadiazole derivatives 2-phenyl-5-aminothiadiazole, 2-cinnamyl-5-aminothiadiazole, and 2-phthalimidomethyl-5-aminothiadiazole (0.01 mol) was refluxed in boiling acetic acid/fused sodium acetate (30 mL/2 g) for 3 h. The solution was poured into an ice / water mixture, stirred and left to settle down affording a white solid. The resulting solid was filtered, washed, dried and finally recrystallized from the proper solvent affording the derivatives **16a-c**.

*5-[2-Ethoxyquinazolone-3-yl]-2-phenylthiadiazole* (**16a**): Brown crystals from DMF; m.p. 172–173 °C; yield 85%. Anal. for C_18_H_14_N_4_O_2_ S (m.w. 350); Found: C, 61.88; H, 4.04; N, 16.08; S, 9.17; Calcd: C, 61.71; H, 4.00; N, 16.00; S, 9.14. IR υ (cm^-1^) 1630 (C=N), 1669 (C=O). MS: *m/z* (int. %) [M+H]^+^ 350 (78.0), 352 (31.1), 191 (100), 193 (23.7), 157 (4.4), 159 (0.1), 175 (49.5), 177 (9.7), 162 (38.2), 164 (3.6), 130 (61.2), 132 (4.1), 103 (1.8), 105 (0.2), 78 (0.7), 80 (0.1); ^1^H-NMR (DMSO-d_6_) δ 1.22 (t, 3H; -OCH_2_*CH_3_*, *J* = 7.1 Hz), 4.4 (q, 2H; -O*CH_2_*CH_3_), 7.41 – 7.94 (m, 5H, Ph-H), 7.44 – 8.20 (m, 4H, quinazolinone).

*5-[2-Ethoxyquinazolone-3-yl]-2-cinnamylthiadiazole* (**16b**): Brown crystals from DMF; m.p. 289-290 °C; yield 85%. Anal. for C_20_H_16_N_4_O_2_ S (m.w. 376); Found: C, 68.99; H, 4.72; N, 16.00; S, 9.03; Calcd: C, 68.97; H, 4.60; N, 16.09; S, 9.20. IR υ (cm^-1^) 1633 (C=N), 1689 (C=O). MS: *m/z* (int. %) [M+H]^+^ 376 (72.0), 378 (14.9), 191 (100), 193 (17.5), 188 (14.2), 190 (3.1), 175 (48.2), 177 (12.7), 157 (5.7), 159 (0.2), 130 (67.1), 132 (1.8), 129 (0.8), 131 (0.2), 122 (1.3), 124 (0.1), 78 (0.6), 80 (0.1); ^1^H-NMR (DMSO-d_6_) δ 1.23 (t, 3H; -OCH_2_*CH_3_*, *J* = 7.1 Hz), 4.43 (q, 2H; -O*CH_2_*CH_3_), 7.42 – 8.20 (4d, 4H, quinazolinone ArH), 7.35 – 7.45 (m, 5H, cinnamyl ArH), 7.20, 7.47 (2d, 2H, *J* = 15.8 Hz, olefinic -H).

*5-[2-Ethoxyquinazolone-3-yl]-2-phthalamidomethylthiadiazole* (**16c**): Brown crystals from DMF; m.p. 303–304 °C; yield 85%. Anal. for C_21_H_15_N_5_O_4_ S (m.w. 433); found: C, 58.72; H, 3.66; N, 16.31; S, 7.42; Calcd: C, 58.20; H, 3.46; N, 16.17; S, 7.39. IR υ (cm^-1^) 1631 (C=N), 1670, 1727, 1776 (3C=O). MS: *m/z* (int. %) [M+H]^+^ 433 (58.0), 435 (22.8), 245 (36.4), 247 (3.4), 191 (100), 193, (56.1), 186 (78.0), 188 (12.7), 175 (30.1), 177 (8.1), 157 (3.2), 159 (0.1), 147 (8.3), 149 (0.3), 130 (48.3), 132 (6.4), 122 (4.5), 124 (0.2), 78 (0.3), 80 (0.1); ^1^H-NMR (DMSO-d_6_) δ 1.21 (t, 3H; -OCH_2_*CH_3_*, *J* = 7.1 Hz), 4.48 (q, 2H; -O*CH_2_*CH_3_), 5.16 (s, 2H; CH_2_, phthalimidomethyl), 7.32–7.86 (m, 4H, quinazol.), 7.94–8.03 (m, 4H, phthalimido).

## Figures and Tables

**Scheme 21:**
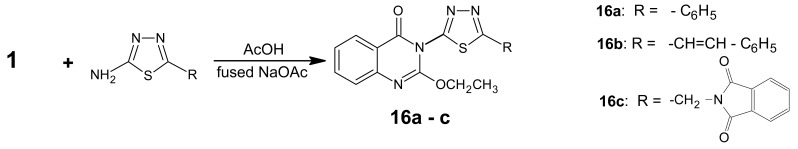
Synthetic pathway for compound **16**.

**Scheme 21:**
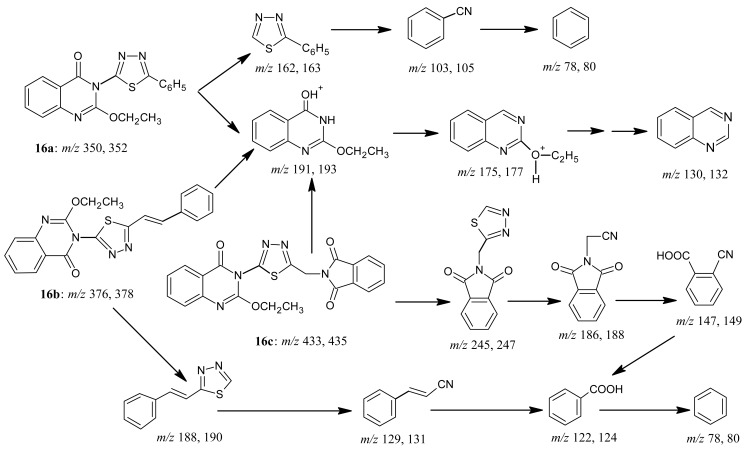
Mass spectra interpretation for compounds **16a-c**.

**Table 1:** *In vivo* antimicrobial activity by agar diffusion method of tested compounds.

**Compound**	**Inhibition zone diameter (mm / mg sample)**				
	*E. coli*	*S. aureus*	*A. flavus*	*C. albicans*	Solvent **control**
Tetracycline	33	31	00	00	----
Amphotericin B	00	00	17	21	----
**3**	12	12	00	12	Chloroform
**6b**	22	17	00	15	Ethanol
6d	15	14	00	00	Ethanol
8	09	12	00	12	Chloroform
9	16	16	12	15	Ethanol
10	10	10	00	00	Chloroform
12	14	15	00	12	Chloroform
14b	13	14	00	00	Ethanol
16a	15	17	13	00	Ethanol
16b	14	15	12	13	Ethanol
16c	09	10	09	10	DMF
